# A Low-Cost GPS GSM/GPRS Telemetry System: Performance in Stationary Field Tests and Preliminary Data on Wild Otters (*Lutra lutra*)

**DOI:** 10.1371/journal.pone.0029235

**Published:** 2012-01-05

**Authors:** Lorenzo Quaglietta, Bruno Herlander Martins, Addy de Jongh, António Mira, Luigi Boitani

**Affiliations:** 1 Department of Biology and Biotechnology “Charles Darwin”, University of Roma ‘La Sapienza’, Roma, Italy; 2 CIBIO - Centro de Investigação em Biodiversidade e Recursos Genéticos, Porto, Portugal; 3 Dutch Otterstation Foundation, Leeuwarden, The Netherlands; 4 ICAAM – Mediterranean Agricultural and Environmental Sciences Institute – University of Évora, Évora, Portugal; Monash University, Australia

## Abstract

**Background:**

Despite the increasing worldwide use of global positioning system (GPS) telemetry in wildlife research, it has never been tested on any freshwater diving animal or in the peculiar conditions of the riparian habitat, despite this latter being one of the most important habitat types for many animal taxa. Moreover, in most cases, the GPS devices used have been commercial and expensive, limiting their use in low-budget projects.

**Methodology/Principal Findings:**

We have developed a low-cost, easily constructed GPS GSM/GPRS (Global System for Mobile Communications/General Packet Radio Service) and examined its performance in stationary tests, by assessing the influence of different habitat types, including the riparian, as well as water submersion and certain climatic and environmental variables on GPS fix-success rate and accuracy. We then tested the GPS on wild diving animals, applying it, for the first time, to an otter species (*Lutra lutra*). The rate of locations acquired during the stationary tests reached 63.2%, with an average location error of 8.94 m (SD = 8.55). GPS performance in riparian habitats was principally affected by water submersion and secondarily by GPS inclination and position within the riverbed. Temporal and spatial correlations of location estimates accounted for some variation in the data sets. GPS-tagged otters also provided accurate locations and an even higher GPS fix-success rate (68.2%).

**Conclusions/Significance:**

Our results suggest that GPS telemetry is reliably applicable to riparian and even diving freshwater animals. They also highlight the need, in GPS wildlife studies, for performing site-specific pilot studies on GPS functioning as well as for taking into account eventual spatial and temporal correlation of location estimates. The limited price, small dimensions, and high performance of the device presented here make it a useful and cost-effective tool for studies on otters and other aquatic or terrestrial medium-to-large-sized animals.

## Introduction

Continuous improvements in component technologies, combined with price reductions for Global Positioning System (GPS) devices is resulting in their increase use for animal tracking [1], [2] and often preferred to traditional very high frequency (VHF) telemetry [3]. GPS technologies indeed improve the efficiency and accuracy of animal locations [4], [5], allowing for more flexibility in sampling design and, often, greater cost-effectiveness in data acquisition [1], [5].

Careful field testing to determine accuracy and potential location biases has always been considered essential for every form of wildlife telemetry [6] and GPS telemetry is no exception [7]–[10]. Researchers have performed several studies with the aim of assessing the effects of habitat type, topography, canopy closure, vegetation structure, cloud cover, day period, fix time interval, and other variables on GPS location acquisition and errors [5]. Nevertheless, as recent as 2010, understanding the causes of GPS errors has been considered a critical need [5].

It is therefore surprising that, to date, the performance of GPS devices used in wildlife telemetry has never been tested specifically in a riparian habitat, despite it being one of the habitat types most commonly used by many animal taxa [11], [12]. The existence of such gap is still more noticeable considering that intense canopy closure, one of the basic constituents of riparian vegetation galleries, has been identified as one of the major causes of GPS failure [4], [5], [13]. Thus, inferences from studies on habitat selection by riparian animals (or animals that intensively use such a habitat type) tagged via GPS devices may yield biased results. Such biases could be even more pronounced in the case of diving animals, as repeated water submersion could interfere with proper GPS functioning. Before they can be applied confidently to diving or riparian animals, GPS technologies should be tested in such a habitat, and preferably under water.

Eurasian otters (*Lutra lutra*) are diving mammals that live almost exclusively in riparian habitats [14], often selecting dense vegetation cover [15], [16]. They seem, therefore, a suitable species on which to test the reliability of GPS tracking systems in riparian habitats, on diving animals, and in dense canopy closure conditions. In addition, this species provides an opportunity to test GPS performance on a medium-sized carnivore, with most previous studies considering larger mammals [17], [18].

Most of the GPS wildlife studies that have been performed so far utilized commercial GPS devices; and, although costs associated with such devices have decreased over the last few years, they typically remain very expensive, limiting their use in projects with budget limitations [10], [17]. Consequently, we developed a low-cost easily constructed GPS GSM/GPRS system (® Dutch Otterstation Foundation, Netherlands) and examined its functioning, under field conditions, in a series of stationary tests performed in Southern Portugal.

The main objective of this study was to test the performance of our GPS device in a riparian habitat and at different depths under water. We, therefore, assessed the effects of three habitat types (including the riparian), canopy closure, and water submersion, as well as the influence of certain climatic variables on the rate of successful localization and accuracy of our GPS system, while also investigating for any potential temporal or spatial correlations in data. In addition, we fitted the device on wild otters (*Lutra lutra*) for the first time to date. This preliminary study allowed us to evaluate our device's performance on free-ranging animals within the habitats they typically use.

## Methods

### Study area

We conducted our study from April 2009 to May 2010 in Alentejo, in Southern Portugal. Portugal is a country where otters are widely distributed [19]. The climate in the study area is typically Mediterranean [20], with an average annual rainfall of 600–700 mm, and rainy days occurring mostly from October to April (http://www.cge.uevora.pt/). Relief is mostly plain with smooth elevations, ranging from 200 to 370 m. The dominant land use is the traditional Mediterranean woodland, designated “montado”, consisting of cork oak (*Quercus suber*) and/or holm oak (*Quercus ilex*) stands combined with extensive agriculture, forestry and livestock grazing [21]. Streams, ponds, and small dams are common, providing an almost continuous water network. The riparian vegetation is dominated by alders (*Alnus glutinosa*), poplars (*Populus nigra*), willows (*Salix atrocinerea*), and brambles (*Rubus spp.*), which provide refuge for otters [22], [23]. Human settlements are concentrated in cities and small villages, with a few farmhouses scattered throughout the landscape.

### Equipment development

As a GPS receiver, we used a GE863-GPS (® Telit, Italy), the smallest (41.4×31.4×3.6 mm) combined GPS Global System for Mobile Communications/General Packet Radio Service (GSM/GPRS) module available in the market at the time of the study, integrating full 20-channel GPS functionality mounted onto a Printed Circuit Board (PCB) (Video S1).

To power the device, we chose a VARTA LiPo battery with 2500 mAh capacity and an estimated average life of 42 days at 4 location records per day (Video S1). To save power, the unit made use of awake–deep sleep cycles [7] on which at each attempt, the unit recorded a location during a 24–380 second period (average = 145.2 seconds; SD = 112.1) and then turned to a low consumption operation mode for the rest of the time until next location attempt.

To the module we connected an encapsulatable GPS antenna for marine and submarine applications (Wi-Sys Communication Inc.) and a PCB GSM antenna (Video S1). Overall dimensions reached approximately 65 mm length×45 mm width×28 mm thickness, for a total weight of 84 g and a price of 630,25 € (excluding VAT).

We coated all components with polyethylene foam and encased them in an enclosure made of a shrinkable tube of Thermoplastic Polyolefin sealed through heat to prevent water penetration (Video S1). We then covered the tube with a thin layer (about 5 mm) of Epoxy Adhesive Super Glue for stronger protection from water, bites, and scrapes against rocks or bushes.

**Figure 1:**
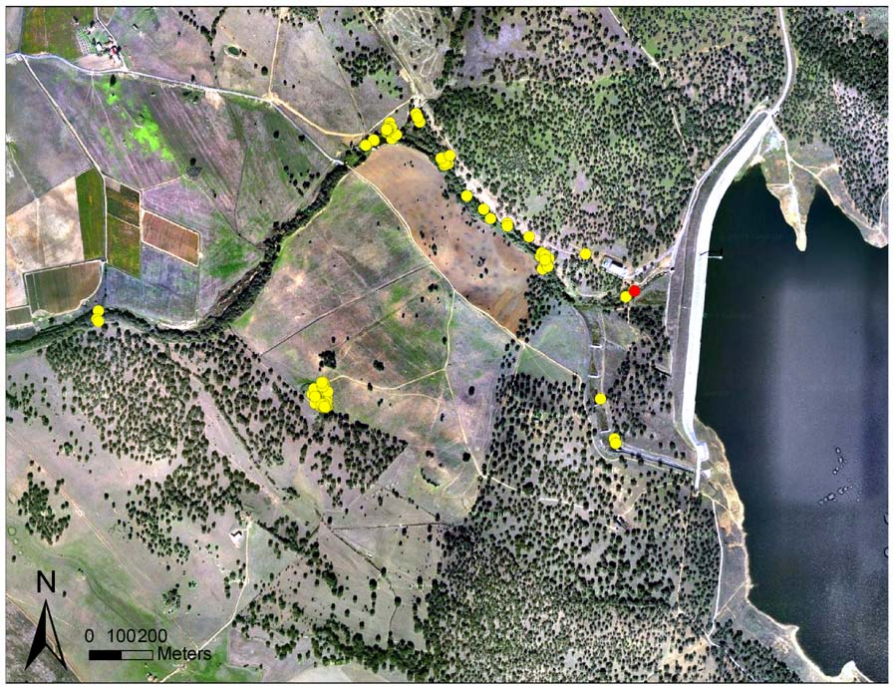
Locations of a wild-free-ranging otter accessed on the Internet in real time. They concerns the GPS harness (H3) (male otter) and are projected on a Google map. Yellow points represent each location; the red point is the capture (and release) site.

GPS devices usually are programmed by researchers to acquire locations according to a requested sampling schedule and battery limits [1]. The Telit module has an onboard Python™ application script interpreter. A GPS data-reporting script using GPRS was produced (® Dutch Otterstation Foundation), with a remote online potential for changing reporting times. We retrieved location data through a combined GSM/GPRS service from a pre-paid SIM card and stored them in an online MySQL database, allowing for visualization of locations directly on Google maps (Fig. 1). The software had a data logger, capable of storing (up to 100) locations whenever the GSM network was not available and sending them during the subsequent reporting attempt with good GSM coverage. We tested two different versions of the GPS software. The second version differed from the first in having a 10-second longer time period for location logging.

Each successful GPS attempt provided the following information: geographic coordinates, date, time, battery state, location status (1D, 2D, and 3D fixes, based upon the number of satellites detected) and the time employed by the unit to retrieve, given a location.

### Field tests

From July to December 2009, we performed a series of field tests. Test sessions lasted, on average, 5.54 h, with 1 location scheduled every 10 or 20 minutes, for a total of 390 attempts. In each session, we placed GPS harnesses at test sites above the ground at roughly the same level as an otter's height. We selected test sites with a purposive convenience sampling criterion [24], stratified on the basis of the different habitat types (Table 1) used by otters monitored (by VHF radio-telemetry) within the framework of a project on the ecology and behaviour of this species at the study site (L. Quaglietta, unpublished data). Test sites located in the middle of dams (on the water surface) were pooled with those located in open woodland, as the former were of limited sample size (N = 24) and both shared almost no vegetation cover.

**Table 1:** Variables used in the evaluation of GPS performance in the stationary tests in Southern Portugal.

Explanatory variables	Type	Description
fSftw	Categorical	GPS software version: (1) older version; (2) newer version
fHabType	Categorical	Habitat types: (1) human settlements; (2) open woodland; (3) riparian
fCanopy2	Categorical	Canopy closure: (1)<60%; (2) 61–100%
fWatSub	Categorical	GPS submersion or emersion: (1) Underwater; (2) Out of water
fDepth	Categorical	Classes of depth at which the GPS was submerged: (1) Out; (2) 0–30 cm; (3) 31–50 cm; (4) 51–150 cm
fLight	Categorical	Daily period: (1) Night (2) Day
fLocal	Categorical	Number code identifying GPS locality
fDate	Categorical	Date of the field test
fInclination	Categorical	Orientation of the GPS: (1) 90°L; (2) 45°; (3) 90°R; (4) 180°
fPosition	Categorical	GPS position relative to the river bed: (1) Near river bank; (2) Center of the river bed
SR	Continuous	Solar radiation (W/m^2^)
W	Continuous	Wind velocity (m/s)
Rain	Continuous	Precipitation (mm)

Response variables	Type	Description
GPS SR	Binary	Scheduled attempts resulting in successful (1) or unsuccessful acquisition (0)
GPS LE	Continuous	Error value of GPS locations comparing with their actual “true” value (m)

The fine temporal scale (10×10 minutes) climatic data were provided by the Geophysics Centre of the University of Évora (http://www.cge.uevora.pt/).

Besides habitat type, during each session we recorded other environmental variables (Table 1) and, specifically for the riparian habitat, GPS inclination towards the sky, GPS position within the riverbed, and underwater depth at which the GPS was submerged (at a mean depth of 0.49±0.26 m, based upon information on otter swimming behavior in the study area – L. Quaglietta, unpublished data). A meteorological station located at the core of the study area furnished some fine temporal-scale climatic data (Table 1).

We defined two types of success rate: the GSM Fix-Success Rate (GSM SR), computed as the proportion of scheduled locations transmitted by the GSM device even if it resulted in no coordinates; and the GPS Fix-Success Rate (GPS SR), corresponding to the proportion of scheduled attempts that resulted in successful location acquisition.

We gave location accuracy, hereafter GPS Location Error (GPS LE), as the Euclidean distance in meters between each individual test location and the “true position” [25], established by a portable GPS receiver (Garmin eTrex® H with high-sensitivity WAAS-enabled). The use of a portable GPS, which is also prone to error, could lead to low accuracy. Unfortunately, we did not have access to a reference station, which could have allowed us to limit such problems by performing a differential correction of the coordinates, as done in other studies [25], [26]. However, we think that our solution may be regarded as acceptable, considering the scope of this study (which was more focused on identifying which environmental variables influence the performance of our GPS system than exactly quantifying the associated errors), the high accuracy displayed by the portable GPS (3.93 m; SD = 1.12; range: 2–7; N = 305) and the very high percentage of 3 D fixes - the most accurate [25], [27], [28], [29] - gathered both in field tests and by wild marked otters (see Results section).

### Data analysis

We modeled the influence of canopy density (fCanopy2), daily period (fLight), habitat type (fHabtype), GPS submersion (fWatSub), software version (fSftw), solar radiation (SR), wind velocity (W), precipitation (Rain) (Table 1) and the interactions between SR and W on GPS SR through a generalized linear mixed-model (GLMM) with a binomial error distribution. We named this model the “Overall acquisition model”.

We evaluated the effects of the same explanatory variables on GPS LE through a Gaussian GLMM, named “Overall accuracy model”. In order to approach normality and stabilize the variances, we transformed (log x + 1) the response variable.

As a second step, we wanted to test the device only in the riparian habitat and using only the last GPS version (the one that, so far, performed better, had the data-logger and that was going to be used later on wild otters). We, therefore, developed two similar models, only including data taken in this habitat type, naming them “Riparian acquisition model” and “Riparian accuracy model”. Besides the original variables, we included three more specific variables: GPS inclination, GPS position, and Depth (Table 1). We transformed (log x + 1) the response variable in the Riparian accuracy model. Moreover, we performed a T-test (equal variances not assumed) on the GPS LE average values taken while the GPS was under and out of water.

We based model selection on the Information Theoretic Approach and performed it through Akaike's Information Criterion (AIC) [30], following the protocol suggested by Zuur et al. [31]. Consequently, we further validated the best model by graphic inspection [31], [32].

The resulting temporal and spatial correlation in the data, that is, the influence of the day and site of the trial on GPS acquisition and accuracy, has been seldom addressed in previous studies (but see Discussion section). To reduce the former, we used the date of field test trials as a random term (Table 1). As for the spatial correlation, we divided the hydrographic network into evenly-spaced (about 2 km) stretches, assigned each GPS test site to a stretch, named fLocal (Table 1), and fitted it also as a random term. Moreover, we used the variable GPS as random term, in order to take into account the variability between each individual GPS used in the field tests. The Riparian accuracy model was more difficult to run without convergence problems, so it was necessary to simplify it to avoid interaction terms [33].

All analyses were performed using R software, version 2.11 (R Development Core Team – 2009 - http://www.R-project.org).

### Otter tagging

Since abdominal implantation of a GPS device is not possible, due to the requirement for an external antenna, implementing this technology on otters must overcome methodological constraints. A standard collar has already been deemed impractical or even risky for otters and it is not recommended for these or other mustelids, because of the similar diameter of their neck and head [14], [34]–[37]. Harnesses seem to be the most reliable alternative for the application of GPS on otters, to date. In fact, they have been already used on otters, and, although some concerns regarding animal welfare and possible risks exist [14], [35], [38], no significant events are reported in the cited studies.

We fitted harnesses with the second software version of the GPS-GPRS device onto six wild otters live-trapped during the already-cited ecological project. Otter trapping and handling procedures followed [38], [39], being in accordance with the guidelines approved by the American Society of Mammalogists for the use of wild mammals in research [40]. The protocol was approved by the Portuguese Institute for Nature and Biodiversity Conservation (license permits N° 104/2009 and 105/2010). Before fitting wild animals, we evaluated the impact of the GPS-harness on a wild-born female otter in captivity, at the Santo André Wildlife Recovery Center (Video S1).

We attached the previously-described case to a thin leather harness (Video S1). For mounting the harnesses on otters, we followed the procedures of two previous studies which deployed VHF-harnesses on the same species [35], [38]. We partly constructed harnesses beforehand, so as to only activate and fit GPSs in the field. This avoided longer animal-handling periods, saved battery power, and allowed for the necessary initial good reception of satellites under open sky.

We added a small VHF radio transmitter to the GPS unit to allow for device retrieval after it dropped off the animal. Drop off was expected to be provoked by wear and tear of the leather's straps. Each harness weighed approximately 220 g, accounting for 4.8% of the weight of the smallest fitted otter and costing approximately 40 € in materials (i.e. leather, tube, glue, tapes). We collected data at a cost of one € per day, independent of the programmed schedule. We scheduled global positioning devices (GPDs) to record locations during nocturnal hours, coinciding with the period of major activity by otters [14], [22], especially in the study area (L. Quaglietta, unpublished data).

When harnesses apparently had dropped off, we made an attempt to recover them, in order to reuse the GPDs, as well as to compute location accuracy in real “ottery” places (Euclidean distance in meters between the “true ottery” position, measured by the portable GPS, and the location recorded by the GPD). We used data from marked otters to compute the overall GSM and GPS SR, as an indication of GPS performance on free-ranging animals.

## Results

### Equipment development and field tests

The final average price of a single GPS-GPRS device, materials, and assembling labor was roughly 790 €.

The wild born otter in captivity appeared to move and behave naturally, paying almost no attention to the GPS harness on its back after the first hour (Video S1). After releasing the animal into the wild, the GPS worked very satisfactorily, yielding a GSM SR of 88% and a GPS SR of 71% (N = 66).

Stationary field-test harnesses successfully acquired locations at the different test sites. Out of the 390 attempts, 263 locations were successfully retrieved by the GSM service, yielding a GSM SR of 67.4%. Satellites were successfully acquired on 239 occasions, giving a GPS SR of 61.2%; within these successful locations, 98.8% were 3D locations and 1.2% 2D. The GPS LE varied widely, considering both software versions used, from 0 to 400 m (mean = 19 m). However, the observed variability in the second version (the one used later on wild otters) was smaller, generating an average GPS LE of 8.94 m (SD = 8.55; range: 0–41 m, with 50% of locations within 6 m and 95% within 27 m; N = 193), much lower than the 60.38 m (SD = 92.23; range: 0–400; N = 45) of the first. GPS LE computed only in the riparian habitat was 10.13 m.

According to the Overall GPS acquisition model, the GPS SR was much higher in human settlements than in the other 2 habitat types (P = 0.015 and 0.008, respectively, for open woodland and riparian). Software version played a very important role (having the highest estimate between the coefficients), with the third version of the software performing significantly better (P = 0.007). Surprisingly, the percentage of canopy had a positive effect on the GPS SR (P = 0.003). Higher solar radiation values were positively associated with the GPS SR (P = 0.009) (Table 2). The model with two random effects (fDate and fLocal) was selected, with fDate accounting for the majority of variability in these data (SD: 1.94), while both AIC values and likelihood ratio test showed no meaning of using GPS as random term. No effects were noticed related to the period of day, wind or precipitation.

**Table 2:** Results of the GLMM model to predict GPS fix-success rate.

Overall acquisition model		
Random effects:		
Groups Name	Std.Dev.	No.
fLocal	1.12	28
fDate	1.94	11
Number of obs:	390	

Fixed effects:	Estimate	Std. Error	P-value
(Intercept)	0.050	1.274	0.9714
fSftw[T.2]	3.522	1.315	0.0074**
fHabType[T.2]	−2.401	0.992	0.0155*
fHabType[T.3]	−2.560	0.966	0.0081**
fCanopy2[T.1]	1.165	0.385	0.0025**
SR	0.002	0.001	0.0088**

The Overall accuracy model (Table 3) revealed a reverse pattern, in terms of habitat type: the GPS LE was lower in open woodland (P = 0.007) and in the riparian zones (P = 0.046) than in human settlements (although, in the latter case, the relationship was only marginally significant). Performance, in terms of accuracy, therefore seemed to be higher in these two habitat types, contrary to what happened with the GPS SR model. The third software update version improved GPS accuracy (P≤0.023). Solar radiation, wind and precipitation did not exert any influence, while nightly hours were positively associated with the GPS LE (P = 0.025). Spatial correlation was limited, having a standard deviation of 0.43. Models with observations nested by individual GPS were selected, with a GPS standard deviation of 0.40. In the Riparian acquisition model, GPS SR was mostly influenced by GPS inclination (whatever the angle was), water submersion (independent of the depth), and, secondarily, by position in the riverbed (Table 4). Indeed, attempts with the device under water, oriented in any position other than horizontal, and located near the river bank significantly worsened GPS SR results. Moreover, high levels of solar radiation seemed to be associated with higher GPS SR (P≤0.001). Also in this case, the model with two random effects (fDate and fLocal) was selected and fDate accounted for most of the variation (SD = 3.68).

**Table 3:** Results of the GLMM model to predict GPS Location Error.

Overall accuracy model		
Random effects:		
Groups Name	Std.Dev.	No.
fLocal	0.43	26
GPS	0.40	4
Number of obs:	238	

Fixed effects:	Estimate	Std. Error	p-value
(Intercept)	3.427	0.432	0.0453*
fSftw[T.2]	−0.635	0.255	0.0233*
fHabType[T.2]	−0.893	0.398	0.0072*
fHabType[T.3]	−0.614	0.376	0.0456*
fLight[T.2]	−0.380	0.157	0.0250*

**Table 4:** Results of the Riparian GLMM model to predict GPS fix-success rate.

Riparian acquisition model		
Random effects:		
Groups Name	Std.Dev.	No.
fLocal	2.84	6
fDate	3.68	5
Number of obs:	216	

Fixed effects:	Estimate	Std. Error	P-value
Intercept	1.146	2.298	0.6180
fPosition[T.1]	−1.797	0.843	0.0331*
fInclination[T.1]	−5.176	1.583	0.0011**
fInclination[T.2]	−5.923	1.929	0.0021**
fInclination[T.3]	−5.753	1.648	0.0005***
fInclination[T.4]	−4.804	1.589	0.0025**
fDeep[T.2]	−4.915	1.518	0.0012**
fDeep[T.3]	−4.120	1.681	0.0143*
fDeep[T.4]	−3.882	1.819	0.0328*
SR	0.016	0.003	<0.0001***

As with the Riparian accuracy model, the GPS LE primarily was negatively affected by water submersion and location within the river bank (Table 5). Both AIC values and likelihood ratio tests of the nested models indicated that the best model was the one with fLocal as the only random term (SD = 0.44).

**Table 5:** Results of the Riparian GLMM model to predict GPS Location Error.

Riparian accuracy model		
Random effects:		
Groups Name	Std.Dev.	No.
fLocal	0.44	5
Number of obs:	137	

Fixed effects:	Estimate	Std. Error	P-value
Intercept	1.857	0.224	0.0000***
fPosition[T.1]	0.462	0.159	0.0053**
fDeep[T.2]	0.530	0.160	0.0000***
fDeep[T.3]	0.783	0.169	0.0000***
fDeep[T.4]	0.653	0.310	0.0354*

Average GPS LE underwater was significantly bigger (mean = 13.37 m; SE = 1.35; N = 41) than the one out of water (mean = 6.58 m; SE = 0.55; N = 96) (t = −4.64; df = 53.56; P = 0.00).

### GPSs mounted on wild otters

We fitted six wild free-ranging otters with GPS-GPRS harnesses (Table 6) (Video S1). All harnesses gathered data, corresponding to a total of 711 locations, for overall GSM and GPS SR of 86.5% and 68.2%, respectively.

**Table 6:** Performance of GPS devices fitted on wild otters in Southern Portugal, in 2009.

Otter		Weight (kg)	Dates of release	NPL	NRL	GSM SR	NSL	GPS SR	3D locations	2D locations	MP (days)
No.	Gender										
H1	F	5.5	1 Oct 09	74	74	100%	54	73.00%	72.20%	27.80%	4
H2	F	4.5	20 Oct 09	221	221	100%	160	72.50%	96.90%	3.10%	10
H3	M	7	14 Dec 09	241	241	100%	192	79.70%	92.70%	7.30%	15
H4	F	6.2	15 Dec 09	120	101	84.20%	92	76.70%	91.30%	8.70%	8
H5	F	6.8	18-mar-10	83	29	34.90%	17	20.50%	76.50%	23.50%	11
H6	F	5.5	11 May 10	45	45	100%	39	86.70%	76.90%	23.10%	6
**Total**				784	711	86.50%	554	68.20%	84.40%	15.60%	54
**TWL**				581	581	100%	445	78.00%	84.70%	15.30%	35

Underlined data refer to the harnesses that had limitations due to software malfunction. TWL = total of the harnesses without limitations. NPL = number of programmed locations. NRL = number of retrieved locations. NSL = number of successfully acquired locations. MP = monitoring period.

Four harnesses worked well (H1, H2, H3 and H6), obtaining all 581 location programmed, giving a GSM SR of 100%. Within the retrieved locations, the positional information was successfully acquired 445 times, yielding an average GPS SR of 78.0%, with 84.7% in 3D and 15.3% in 2D (Table 6). The remaining 2 harnesses (H4 and H5), despite having retrieved 130 of the 203 programmed locations (average GPS SR = 48.6%; 83.9% were 3D and 16.1% 2D), did not transmit at the requested frequency, but instead at irregular times, because of software malfunctioning. These locations, however, still seemed highly accurate, as points were never located far from watercourses (Fig. 1).

The monitoring period for each animal averaged 9 days, ranging from 4 to 15 (Table 6). Four harnesses (H1, H3, H5 and H6) were recovered from the field. Their accuracy was, on average, within 4.3 m. Recovered harnesses revealed good condition of the transmitter case (only a few minor scratches) with ventral and dorsal straps weakened by water and abrasion, suggesting easy release by the otters. Harness H2 was recovered with the carcass of the animal, killed by humans as revealed by necropsy (L. Quaglietta, unpublished data). The stomach was full of food and the general condition of the animal was good, suggesting that the harness did not have an impact upon its death. The harness was still in good condition and well-deployed on the otter's body; consequently, it likely would have remained attached for a few more days.

**Figure 2:**
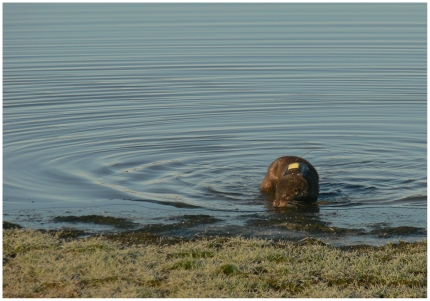
Wild free-ranging female otter (*Lutra lutra*) fitted with a GPS harness after the release.

The female marked with harness 5 was recaptured after she dropped off the device. Because of this, it was possible to document that her body showed no signs of previous injuries and that she was lactating. Based upon the scant existing information on wild otter gestation periods [14] and on the time passed between captures, we estimated that this animal reproduced while still with the harness or immediately after it fell off. Animals fitted with GPS harnesses (Fig. 2) showed space use patterns similar to those of otters monitored by VHF telemetry in the same project and area (L. Quaglietta, unpublished data) and were observed while swimming and moving with no apparent restrictions on their movements (pers. obs.).

## Discussion

The wide applicability and benefits of GPS technologies in wildlife telemetry are increasingly cited in the literature [2], [9]. However, most GPS functioning tests have examined devices produced industrially and designed for large mammals [17]. Moreover, GPS functioning has never been previously tested on diving freshwater animals or in a riparian habitat; and, to our knowledge, only a few studies have evaluated the potential effects of climatic variables (see Introduction).

Here, we have presented a manufactured GPS-GPRS device that is affordably priced (similar to another low-cost GPS system recently used on pampas deer - [17]); and we provide information on its performance in riparian as well as other habitat types, and on wild otters.

### Field tests

The GSM and GPS Fix-Success Rates of our GPS-GPRS system are well within the range reported by other authors who have used commercial GPS radio-collars [1], [7], [41].

The higher amount of logging time provided with the second software update was very effective, leading to an average GPS LE of 8.9 m. This result is in accordance with previous studies that identified a positive relationship between logging duration and GPS performance [42]. Importantly, GPS accuracy was high even if computed only in the riparian habitat. Such error values are lower than the mean of most of those reported in GPS wildlife-tracking studies, which is roughly 10–30 m [4], [5], [13], [25], [26], [43], [44]. The remarkable frequency of 3D locations, which are normally related to higher accuracy [13], [25], [27], [29], seems to confirm the high reliability and accuracy of our GPD. In addition, a visual examination of the locations collected in the riparian habitat, both in field tests and by tagged otters, revealed that these were always located within the narrow (10 m) band of riparian vegetation of the sampled rivers, thereby appearing quite accurate (Fig. 1).

Unlike other studies, where canopy density has been reported to significantly reduce the rate of successful location acquisition attempts [45], [46], we failed to detect any sound influence of this variable; and in our Overall acquisition model, denser canopy categories even exerted a positive effect upon GPS SR (but see text below for a discussion of the possible negative effects of dense canopy).

Several authors have reported that habitat characteristics may significantly reduce location acquisition [13], [27], [47]. In our study, open woodlands and riparian habitats were associated with more accurate locations than human settlements (although the p-value concerning the relationship with riparian habitat only approached significance), which is a very unexpected and reassuring result for a GPS that has to be used on riparian animals. A possible explanation is that human settlements may be more susceptible to different types of interference, and these may have played a role in the process of location logging, thereby lowering accuracy. On the other hand, urban areas exhibited higher acquisition rates than the other two habitat types, possibly because, in the former habitat, GPDs remained in more flat and stable conditions, which may have had a positive influence upon satellite acquisition. The high values of solar radiation, which were associated with higher GPS SR, are normally related to clear skies which, in turn, could have allowed for better satellite availability. In this regard, a positive relationship between sky availability and GPS functioning has been documented already [26], [42]. However, the very small coefficient estimate value of the solar radiation suggests care in the interpretation of the effect of this variable.

As concerns riparian models, GPS SR and LE largely were affected by water submersion (independent of depth) and, secondarily, by GPS position within the riverbed. The negative effect of water submersion, both on the probability of localization and location accuracy, is not surprising. So far, and to the best of our knowledge, no previous study has tested GPS devices under freshwater conditions, so no comparisons are available. However, since otters are not completely submerged most of the time (they mainly plunge during predatory activities) [14] (pers. obs.), the error due to submersion may not be such a strong limitation as is often noted for marine mammal GPS tracking [48], [49]. This conjecture is further supported by the high success rate achieved when the GPS was mounted on wild free-ranging otters (see next section). The explanation regarding the position within the riverbed may strengthen the call for caution concerning the potential negative effects of very dense canopies. Indeed, when GPDs were placed on the river bank, they were between roots, or under dense shrubs and rocks, simulating usual otter resting habitats [14]. Consequently, these elements may have constituted a more robust barrier to satellite availability than the taller arboreal riparian vegetation, which did not exhibit a negative effect in the Overall Accuracy model and even exerted a positive effect in the Overall Acquisition model.

The inclination of the GPS, whatever the angle, promoted a lower GPS SR, in accordance with what was reported by [44] and identified in other studies [5]. This can lead to missing locations, since GPS inclination may occur frequently during an animal's normal activities [27]. However, it is worth noting that GPS LE was not influenced by this variable and that the GPS SR of wild otters was even higher than that obtained in the field tests (see Results and the next section), contrary to what occurred in other studies [8], [33]. Solar radiation also, in this case, seemed to have a positive effect on gathering the signal, possibly confirming the importance of clear weather.

Thus, constraints in tracking otters or other aquatic mammals with the presented GPS device mainly seem related to the amount of time animals spend under water and, secondarily, to the frequency with which they are near the river bank, instead of at the center of the riverbed, or burrowed in dens. On this regard, no locations were acquired by a GPD fitted on a wild free-ranging otter (H2) when the latter was inside a hole in the ground (pers. obs.). It is therefore highly suggested to avoid localization trials during the period of otter inactivity.

Random effects accounted for some variability in the datasets, in every model, suggesting that the particular day (and, secondarily, site) in which the tests were done may have played a role and that there was some slight variability in the performance between the individual GPDs. Ignoring temporal and spatial correlations can, therefore, lead to biased results. Hence, we suggest that researchers take these factors into account when they evaluate GPSs in stationary tests, as only rarely done (but see [50]), adapting to what instead is frequently done in habitat selection studies [51].

### GPS devices mounted on wild otters

GPS harnesses on wild otters performed very well, as overall GPS SR was indeed higher than in field tests and a very high frequency of 3D locations (84.4%) was obtained. These results are surprising, given that GPS SRs from free-ranging animals traditionally have been lower than those obtained during stationary tests [5], [25], [33] and that otters used the same habitats that we used for the stationary tests.

Other authors who previously used harnesses on otters have expressed some concerns because of potential deleterious effects upon the animals themselves, both related to friction from the harness material and the risk of the harness becoming entangled under water [35], [38]. In our case, no injuries related to the equipment were noted for any monitored otter. On the contrary, we documented one female otter actually lactating shortly after the monitoring period with the harness. Moreover, the fur of this recaptured female revealed no signs of abrasion from the harness.

At this stage, the applicability of our GPS telemetry system may be primarily limited by the harness' weight/dimensions and retention times. Indeed, although the equipment was lighter than most previously-used devices [17] and weighed less than 5% of the fitted otter's body mass, as generally recommended [52], the harnesses still were not appropriate for cubs/young otters or other animals weighing less than 4 kg. Its application on smaller animals (up to 2–3 kg) remains possible, however, albeit with modifications (e.g. collars, which are smaller and lighter than the harnesses). Our retention times were quite inferior to those obtained in previous studies of harnessed otters [35], [38], in which retention times averaged 30–40 days and extended as long as 98 days. Such a big difference may be explained partially by a difference in harness dimensions (our GPS harness was larger and this could have produced more friction and consequent wear), and our probably exaggerated concern about securing the harnesses tightly. However, an ongoing study in Ireland is showing that obtaining longer durations is possible for otters using GPS harnesses, as otters marked with GPS harnesses very similar to ours already have furnished data up to 40 days (Ó Néill and De Jongh, personal communication).

### Management implications

Based upon the results of this study, we propose a reliable and low-cost method of GPS telemetry that seems to be reliable in riparian habitats and diving animals. Demonstrating a low average error, the tool seems to be suitable for a wide range of studies [2], [5], [45], especially in short-term research for fine temporal-spatial scale resource use, monthly home-ranges, activity patterns, and social interactions, among others. With a very convenient trade-off between price and quality, it represents a useful and cost-effective alternative to available commercial GPS devices for research on otters and many other medium to large sized terrestrial and diving mammals (as well as on smaller animals through the use of collars).

Moreover, GPS biases (affecting both accuracy and success rate) appear to be affected by many other factors, which are too numerous to identify at this time [5]. This highlights the need to undertake more site-specific pilot studies [6], aimed at addressing and identifying the main factors affecting GPS performance in specific study areas, habitat types and animals, in order to properly correct for GPS errors and missing locations in posterior habitat selection studies [5].

## Supporting Information

Video S1 Videos of GPS assembling and fitting on an otter in captivity and a GPS-harnessed wild free-ranging otter.
(WMV)

## References

[1] Rodgers AR, Boitani L, Fuller TK (2000). Recent Telemetry Technology.. Research techniques in animal ecology. Controversies and consequences.

[2] Tomkiewicz SM, Fuller MR, Kie JG, Bates KK (2010). Global positioning system and associated technologies in animal behavior and ecological research.. Philos T Roy Soc B.

[3] Pellerin M, Saïd S, Gaillard JM (2008). Roe deer *Capreolus capreolus* home-range sizes estimated from VHF and GPS data.. Wildlife Biol.

[4] Hulbert I, French J (2001). The accuracy of GPS for wildlife telemetry and habitat mapping.. J Appl Ecol.

[5] Frair JL, Fieberg J, Hebblewhite M, Cagnacci F, DeCesare NJ (2010). Resolving issues of imprecise and habitat-biased locations in ecological analyses using GPS telemetry data.. Philos T Roy Soc B.

[6] Withey JC, Bloxton TD, Marzluff JM, Millspaugh JJ, Marzluff JM (2001). Effects of Tagging and Location Error in Wildlife Radiotelemetry Studies.. Radio Tracking and Animal Populations.

[7] Gau RJ, Mulder R, Ciarniello LJ, Heard DC, Chetkiewicz CLB (2004). Uncontrolled field performance of Televilt GPS-Simplex TM collar on grizzly bears in western and northern Canada.. Wildlife Soc B.

[8] Graves TA, Waller JS (2006). Understanding the causes of missed global positioning system telemetry fixes.. J Wildlife Manage.

[9] Cagnacci F, Boitani L, Powell RA, Boyce MS (2010). Animal ecology meets GPS-based radiotelemetry: a perfect storm of opportunities and challenges.. Philos T Roy Soc B.

[10] Hebblewhite M, Haydon DT (2010). Distinguishing technology from biology: a critical review of the use of GPS telemetry data in ecology.. Philos T Roy Soc B.

[11] Rondinini C, Ercoli V, Boitani L (2006). Habitat use and preference by polecats (*Mustela putorius* L.) in a Mediterranean agricultural landscape.. J Zool.

[12] Matos HM, Santos MJ, Palomares F, Santos-Reis M (2009). Does riparian habitat condition influence mammalian carnivore abundance in Mediterranean ecosystems?. Biodivers Conserv.

[13] Di Orio AP, Callas R, Schaefer RJ (2003). Performance of two GPS telemetry collars under different habitat conditions.. Wildlife Soc B.

[14] Kruuk HA, Kruuk HA (2006). Otters: ecology, behaviour, and conservation.

[15] Green J, Green R, Jefferies DJ (1984). A radio-tracking survey of otters *Lutra lutra* on a Pertshire river system.. Lutra.

[16] Durbin LS (1998). Habitat selection by five otters *Lutra lutra* in rivers of northern Scotland.. J Zool.

[17] Zucco CA, Mourão G (2009). Low-Cost Global Positioning System Harness for Pampas Deer.. J Wildlife Manage.

[18] Mattisson J, Andrén H, Persson J, Segerstrom P (2010). Effects of Species Behavior on Global Positioning System Collar Fix Rates.. J Wildlife Manage.

[19] Trindade A, Farinha N, Florêncio E (1998). A distribuição da Lontra *Lutra lutra* em Portugal - situação em 1995. Estudos de Biologia e Conservação da Natureza, 28.

[20] Carmel Y, Flather CH (2004). Comparing landscape scale vegetation dynamics following recent disturbance in climatically similar sites in California and the Mediterranean basin.. Landscape Ecol.

[21] Pinto-Correia T, Ribeiro N, Sá-Sousa P (2011). Introducing the *montado*, the cork and holm oak agroforestry system of Southern Portugal.. Agroforest Syst.

[22] Beja P (1996). Temporal and spatial patterns of rest-site use by four female otters *Lutra lutra* along the south-west coast of Portugal.. J Zool.

[23] Mason CF, Macdonald SM (1986). Otters: ecology and conservation.

[24] Thompson WL, White GC, Gowan C (1998). Monitoring Vertebrate Populations.

[25] Cargnelutti B, Coulon A, Hewison AJM, Goulard M, Angibault JM (2007). Testing Global Positioning System performance for wildlife monitoring using mobile collars and known reference points.. J Wildlife Manage.

[26] Sager-Fradkin KA, Jenkins KJ, Hoffman RA, Happe PJ, Beecham JJ (2007). Fix Success and Accuracy of Global Positioning System Collars in Old-Growth Temperate Coniferous Forests.. J Wildlife Manage.

[27] Moen R, Pastor J, Cohen Y, Schwartz CC (1996). Effects of moose movement and habitat use on GPS collar performance.. J Wildlife Manage.

[28] Dussault C, Courtois R, Ouellet JP, Huot J (2001). Influence of satellite geometry and differential correction on GPS location accuracy.. Wildlife Soc B.

[29] Rempel RS, Rodgers AR (1997). Effects of differential correction on accuracy of a GPS animal location system.. J Wildlife Manage.

[30] Burnham KP, Anderson DR, Burnam KP, Anderson DR (2002). Model selection and multimodel inference: a practical information-theoretic approach. Second Edition.

[31] Zuur AF, Ieno EN, Walker N, Saveliev AA, Smith GM, Gail M, Krickeberg K, Samet J, Tsiatis A, Wong W (2009). Mixed effects models and extensions in ecology with R.

[32] Zuur AF, Ieno EN, Smith GM, Gail M, Krickeberg K, Samet J, Tsiatis A, Wong W (2007). Analysing Ecological Data.

[33] Schwartz CC, Podruzny S, Cain SL, Cherry S (2009). Performance of Spread Spectrum Global Positioning System Collars on Grizzly and Black Bears.. J Wildlife Manage.

[34] Melquist WE, Hornocker MG (1979). Methods and techniques for studying and censuring river otter populations..

[35] Mitchell-Jones AJ, Jefferies DJ, Twelves J, Green J, Green R (1984). A practical system of tracking otters using radiotelemetry and Zn65.. Lutra.

[36] Zschille J, Stier N, Mechthild R (2008). Radio tagging American Mink (*Mustela vison*) - experience with collar and intraperitoneal implanted transmitters.. Eur J Wildlife Res.

[37] Melquist WE, Hornocker MG (1983). Ecology of river otters in west central Idaho.. Wildlife Monogr.

[38] Ó Néill L, Wilson P, de Jongh AWJJ, de Jong Tj, Rochford J (2008). Field techniques for handling, anaesthetising and fitting radio-transmiters to Eurasian otters (*Lutra lutra*).. Eur J Wildlife Res.

[39] Ó Néill L, de Jongh AWJJ, Ozolinš J, de Jong Tj, Rochford J (2007). Minimizing Leg-hold Trapping Trauma for Otters with Mobile Phone Technology.. J Wildlife Manage.

[40] Gannon WL, Sikes RS, and the Animal Care and Use Committee of the American Society of Mammalogists (2007). Guidelines of the american society of mammalogists for the use of wild mammals in research.. J Mammal.

[41] D'Eon RG, Serrouya R (2005). Mule deer seasonal movements and multiscale resource selection using global positioning system radiotelemetry.. J Mammal.

[42] Hansen MC, Riggs RA (2008). Accuracy, Precision, and Observation Rates of Global Positioning System Telemetry Collars.. J Wildlife Manage.

[43] Lewis JS, Rachlow JL, Garton EO, Vierling LA (2007). Effects of habitat on GPS collar performance: using data screening to reduce location error.. J Appl Ecol.

[44] D'Eon RG, Delparte D (2005). Effect of radio-collar position and orientation on GPS radio-collar performance, and the implications of PDOP in data screening.. J Appl Ecol.

[45] Frair JL, Nielson SE, Merrill EH, Lele SR, Boyce MS (2004). Removing GPS collar bias in habitat selection studies.. J Appl Ecol.

[46] Heard DC, Ciarniello LM, Seip DR (2008). Grizzly Bear Behavior and Global Positioning System Collar Fix Rates.. J Wildlife Manage.

[47] Rempel RS, Rodgers AR, Abraham KF (1995). Performance of a GPS animal location system under boreal forest canopy.. J Wildlife Manage.

[48] Sisak M (1998). Animal-borne GPS and the deployment of a GPS based archiving datalogger on Hawaiian Monk Seal (*Monachus schauinslandi*).. Mar Technol Soc J.

[49] Jay CV, Garner GW (2002). Performance of a satellite-linked GPS on Pacific walruses (*Odobenus rosmarus divergens*).. Polar Biol.

[50] Dennis TE, Chen WC, Koefoed IM, Lacoursiere CJ, Walker MM (2010). Performance Characteristics of Small Global-Positioning-System TrackingCollars for Terrestrial Animals.. Wildl Biol Pract.

[51] Fieberg J, Matthiopoulos J, Hebblewhite M, Boyce MS, Frair JL (2010). Correlation and studies of habitat selection: problem, red herring or opportunity?. Philos T Roy Soc B.

[52] Aldridge HDJN, Brigham RM (1988). Load carrying and maneuverability in an insectivorous bat: A test of the 5% “rule” of radio-telemetry.. J Mammal.

